# Estimating the phase volume fraction of multi-phase steel via unsupervised deep learning

**DOI:** 10.1038/s41598-021-85407-y

**Published:** 2021-03-15

**Authors:** Sung Wook Kim, Seong-Hoon Kang, Se-Jong Kim, Seungchul Lee

**Affiliations:** 1 Department of Mechanical Engineering, Pohang University of Science and Technology, 77 Cheongam-ro, Pohang, Republic of Korea; 2 Korea Institute of Materials Science, 797 Changwon-daero, Seongsan-gu, Changwon, Republic of Korea; 3 Graduate School of Artificial Intelligence, Pohang University of Science and Technology, 77 Cheongam-ro, Pohang, Republic of Korea; 4 Institute of Convergence Research and Education in Advanced Technology, Yonsei University, 50 Yonsei-ro, Seoul, Republic of Korea

**Keywords:** Metals and alloys, Mechanical engineering, Characterization and analytical techniques

## Abstract

Advanced high strength steel (AHSS) is a steel of multi-phase microstructure that is processed under several conditions to meet the current high-performance requirements from the industry. Deep neural network (DNN) has emerged as a promising tool in materials science for the task of estimating the phase volume fraction of these steels. Despite its advantages, one of its major drawbacks is its requirement of a sufficient amount of training data with correct labels to the network. This often comes as a challenge in many areas where obtaining data and labeling it is extremely labor-intensive. To overcome this challenge, an unsupervised way of learning DNN, which does not require any manual labeling, is proposed. Information maximizing generative adversarial network (InfoGAN) is used to learn the underlying probability distribution of each phase and generate realistic sample points with class labels. Then, the generated data is used for training an MLP classifier, which in turn predicts the labels for the original dataset. The result shows a mean relative error of 4.53% at most, while it can be as low as 0.73%, which implies the estimated phase fraction closely matches the true phase fraction. This presents the high feasibility of using the proposed methodology for fast and precise estimation of phase volume fraction in both industry and academia.

## Introduction

Automotive steel products are required to have good mechanical properties with high toughness and strength, which are mainly accomplished by controlling the distributions of micro-constituents in steels. Numerous studies have investigated the formulation of various steel microstructures based on parameters such as temperature, the concentration of carbon, time, and thermomechanical processing, including heat treatments, cooling, annealing, etc. Nonetheless, only a few studies have been successful in the identification and quantification of microstructures, which is of paramount interest in the steel-making industry and academia. The separation of phases becomes more challenging and ambiguous, as the number of micro-constituents gets larger due to the complex characteristics of microstructures under different conditions. For example, complex micro-constituents can often possess the same crystallographic arrangement but with varying degrees of defects in the cells^1^, which makes it difficult to distinguish between themselves.

The basic form of phase identification as well as the estimation of phase volume fraction consists of manually counting the appearances of each micro-constituent through optical microscopy (OM) or scanning electron microscopy (SEM). The so-called point counting methodology is often used as a reference in the literature^1–4^ for verifying if a proposed quantification method is appropriate. Even though it generally assures high accuracy of the actual phase volume fraction, it is often limited in its usage for the enormous amount of time and a large number of images necessary to obtain a 95% reliability^4,5^.

Electron backscatter diffraction (EBSD) has recently become a widely used tool to make microstructural classification of steels^1,2,6–8^. EBSD offers several features, including image quality that allows us to show detailed features of the microstructures such as the boundaries. It also allows phase identification by phase contrast presented from different diffraction intensities of each phase. As such, we also utilize EBSD images in this study to extract raw training data. Wilson et al.^9^ distinguished martensite from ferrite simply using a threshold pattern quality (PQ) value. Pixels with lower PQ than the threshold were classified as martensite, in another case as ferrite. However, the method is valid only when the PQ profile exhibits a clear bimodal distribution. Kang et al.^1^ proposed applying a grain-average function to process the PQ profile and unveil the dominant distribution peaks. The author suggests extracting martensite from a ferrite matrix in DP steels using the average band contrast and identifying bainite of TRIP steel with the local variations of band contrast and orientation inside a grain. However, the study emphasizes the dependency of phase volume fraction on the user-defined definition angle of grain boundary, which is the case for most EBSD-based identifications. Tomaz et al.^2^ presented an almost identical phase fraction estimation on a low manganese HTP steel utilizing EBSD with a maximum fraction difference of 5%, but the author also acknowledges criteria for separation would vary for different types of steels and processing parameters.

Not only are EBSD-based methods unreliable for different types of steel and processing parameters, but they are also labor-intensive. In recent years, such a disadvantage led to the development of numerous data-driven techniques and studies^3,10–13^ along with the rise of machine learning. Gola et al.^11^ used a few data mining methods such as standardization and successive backward elimination to pre-process raw data and made classification by nonlinear SVM. The study achieved the classification accuracy of 87.15% on dual-phase steel consisting of ferrite and martensite when used with feature elimination and standardization. Azimi et al.^12^ first demonstrated an automated EBSD image labeling scheme using the deep learning-based image segmentation method reaching the state-of-the-art classification accuracy of 93.94%. Bulgarevich et al.^13^ similarly demonstrated an automated optical microscopy steel image labeling at pixel-level using a fast Random Forest statistical algorithm, which showed a high percentage and location areas agreed between machine learning and manual examination results. Though it is difficult to tell which result is the best due to unmatched test data and criteria for evaluation, high accuracy was guaranteed only if there had been substantial manually labeled training data. In this sense, the ease of implementation has not been enhanced significantly for data-driven methods than the traditional methods for the estimation of phase volume fraction.

Therefore, we propose a novel means of estimating the phase volume fraction of multi-phase steel without the manual labeling of phases, following an unsupervised manner. This is demonstrated by using a type of generative model, InfoGAN to generate points of specific labels, followed by the training of Multi-layer Perceptron (MLP) classifier using the generated sets. InfoGAN is an extension to vanilla generative adversarial network (GAN) that allows for disentangled latent representation learning by training given input samples along with codes. The disentangled latent representation allows for users’ control because unlike GAN, users can expect what samples would be generated by controlling the codes to certain directions. To validate that our proposed model is applicable for a wide range of AHSS alloys, six different types of steels with varying compositions of microstructures are made and tested on it. The result shows that regardless of the chemical compositions, the model estimates the phase volume fraction very well with the mean relative error of 0.73% at best. As far as we are concerned, this is the first study on the quantification of multi-phase steel based on unsupervised deep learning.

The rest of the paper is broken down as follows. “Revision of recent generative deep learning techniques” provides a recap on the related deep learning techniques encountered in this study whilst “Methodology” details the proposed methodology. The experimental result is discussed in “Results and discussion” and finally, the paper is concluded in “Conclusion”.

## Revision of recent generative deep learning techniques

### Generative adversarial network (GAN)

Since its first advent as a novel framework of a generative model, GAN^14^ has stimulated an explosion of related works in the deep learning community especially for generating realistic images of human beings, animals, objects, and backgrounds. GAN is known to learn data distributions implicitly, which is why it can often be very powerful in imitating the distributions. It is composed largely of two components, a generative model $G$ that captures the data distribution and a discriminator $D$ that figures out whether or not a sample came from the training data. GAN is frequently referred to as a minimax game since the two models compete against each other for simultaneous optimization. Both models are usually differentiable multilayer perceptrons. The ultimate goal of the algorithm is to reach the equilibrium state in which the probability of $D$ is equal to 0.5. The objective function is as follows:

1 $$\underset{G}{\mathrm{min}} \, \underset{D}{\mathrm{max}}V\left(D, G\right)={E}_{x\sim {{P}}_{data}(x)}[\mathrm{log}(D\left(x\right))]+{E}_{z\sim {P}_{z}(z)}[\mathrm{log}(1-D(G(z)))]$$

As few weaknesses of GAN (e.g., mode collapse) were reported, numerous variants of GAN have been introduced in addition to various training techniques^15^ that made it possible to solve the issues. Information maximizing generative adversarial network (InfoGAN) is one that fixes the problem of learning entangled latent representation.

### Information maximizing generative adversarial network (InfoGAN)

InfoGAN^16^ is an extension to GAN, which can learn disentangled latent representation in an unsupervised manner. Disentangled representation allocates a separate set of dimensions for each salient attribute that is informative for distinguishing data of different categories. Learning in such a way brings an advantage over the traditional GAN in that it can control what to output. This is made possible by having an extra term in the objective function, which maximizes the mutual information between an observation and a latent variable during training. InfoGAN decomposes the input noise vector into two parts that are denoted by $z$, a source of noise, and $c$, a latent code. The latent code $c$ represents the salient attributes or the semantic features of the data distribution. The objective function of InfoGAN is as follows:

2 $$\underset{G}{\mathrm{min}} \, \underset{D}{\mathrm{max}}V\left(D, G\right)=V\left(D, G\right)- \lambda I(c, G(z, c))$$

Mutual information between the latent code $c$ and the generated sample is denoted by $I(c, G(z,c))$. The intuitive interpretation of mutual information is the reduction of uncertainty in $c$ when $G(z,c)$ is observed. By maximizing it, the model will be trained so that $c$ and generated samples are relevant to each other. In reality, however, the computation of the term is costly because of posterior $P(c|x)$^16^. suggests estimating it using a lower bound by replacing the posterior with an auxiliary distribution $Q(c|x)$ for approximation, a technique known as Variational Information Maximization^17^.

3 $$\begin{aligned} I\left( {c,{ }G\left( {z, c} \right)} \right){ } = & H\left( c \right) - H(c|G\left( {z, c} \right)) \\ = & E_{{x\sim G\left( {z, c} \right)}} \left[ {E_{{c^{\prime}\sim P(c|x)}} [logP(c^{\prime}|x)]} \right] + H\left( c \right) \\ = & E_{{x\sim G\left( {z, c} \right)}} \left[ {D_{KL} \left( {P\left( { \cdot {|}x} \right) || Q\left( { \cdot {|}x} \right)} \right) + E_{{c^{\prime}\sim P(c|x)}} \left[ {logQ(c^{\prime}|x)} \right]} \right] + H\left( c \right) \\ \ge & E_{{x\sim G\left( {z, c} \right)}} \left[ {E_{{c^{\prime}\sim P\left( {c{|}x} \right)}} \left[ {logQ\left( {c^{\prime}{|}x} \right)} \right]} \right] + H\left( c \right) \\ \end{aligned}$$

By treating $H(c)$ as a constant and using the formula ${E}_{x\sim X, y\sim Y|x}\left[f(x, y)\right]={E}_{x\sim X, y\sim Y|x, {x}^{^{\prime}}\sim X|y}[f({x}^{^{\prime}}, y)]$, a variational lower bound ${L}_{I}(G, Q)$ of mutual information is defined as follows:

4 $$\begin{aligned} L_{I} \left( {G, Q} \right){ } = & E_{{c\sim P\left( c \right), x\sim G\left( {z, c} \right)}} \left[ {logQ(c|x)} \right] + H\left( c \right) \\ = & E_{{x\sim G\left( {z, c} \right)}} \left[ {E_{{c^{\prime}\sim P(c|x)}} [logQ(c^{\prime}|x)]} \right] + H\left( c \right) \\ \le & I\left( {c,{ }G\left( {z, c} \right)} \right) \\ \end{aligned}$$

In Eq. (3), it is notable that as the auxiliary distribution $Q$ becomes similar to the true distribution $P$, the lower bound becomes closer to the mutual information term. Hence, the maximal mutual information is achieved when the lower bound attains its maximum ${L}_{I}\left(G, Q\right)=H(c)$. To conclude, the objective function of InfoGAN can be rewritten as follows:

5 $$\underset{G,Q}{\mathrm{min}} \, \underset{D}{\mathrm{max}}{V}_{InfoGAN}\left(D, G, Q\right)=V\left(D, G\right)-\lambda {L}_{I}(G, Q)$$

The intuition behind the InfoGAN model is to find an auxiliary distribution $Q$ that changes $G(z,c)$ to the right $c$ and at the same time, a generator $G$ that generates the right $G(z,c)$ so that $Q$ operates well.

## Methodology

### General workflow

**Figure 1:**
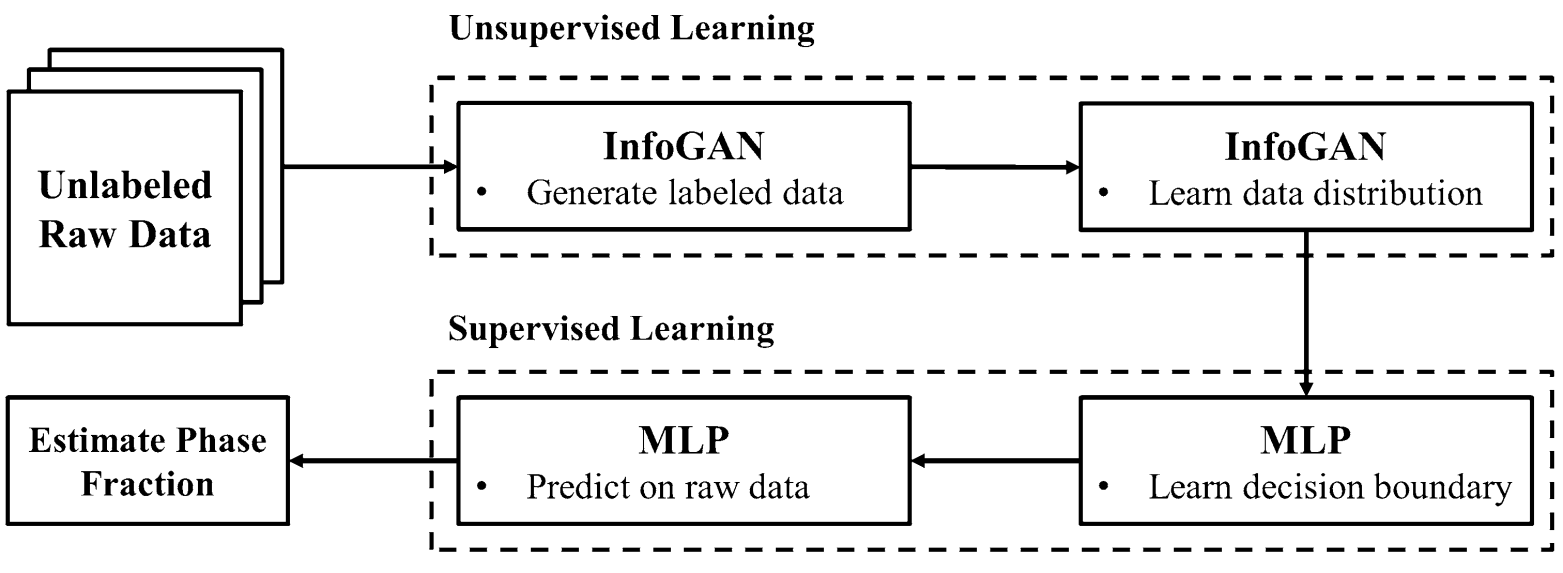
The workflow of the proposed method.

The overall workflow of the proposed method is illustrated in Fig. 1. It is largely divided into two parts. The first part in which InfoGAN is trained using unlabeled raw data is named unsupervised learning. This corresponds to Phase I of Fig. 5. The next part in which an MLP classifier is trained to learn a decision boundary and predict labels on the raw data is named supervised learning. This corresponds to Phase II of Fig. 5. Lastly, the phase volume fraction is estimated by summing up the areas of the identically labeled samples.

### Estimation of phase volume fraction

**Figure 2:**
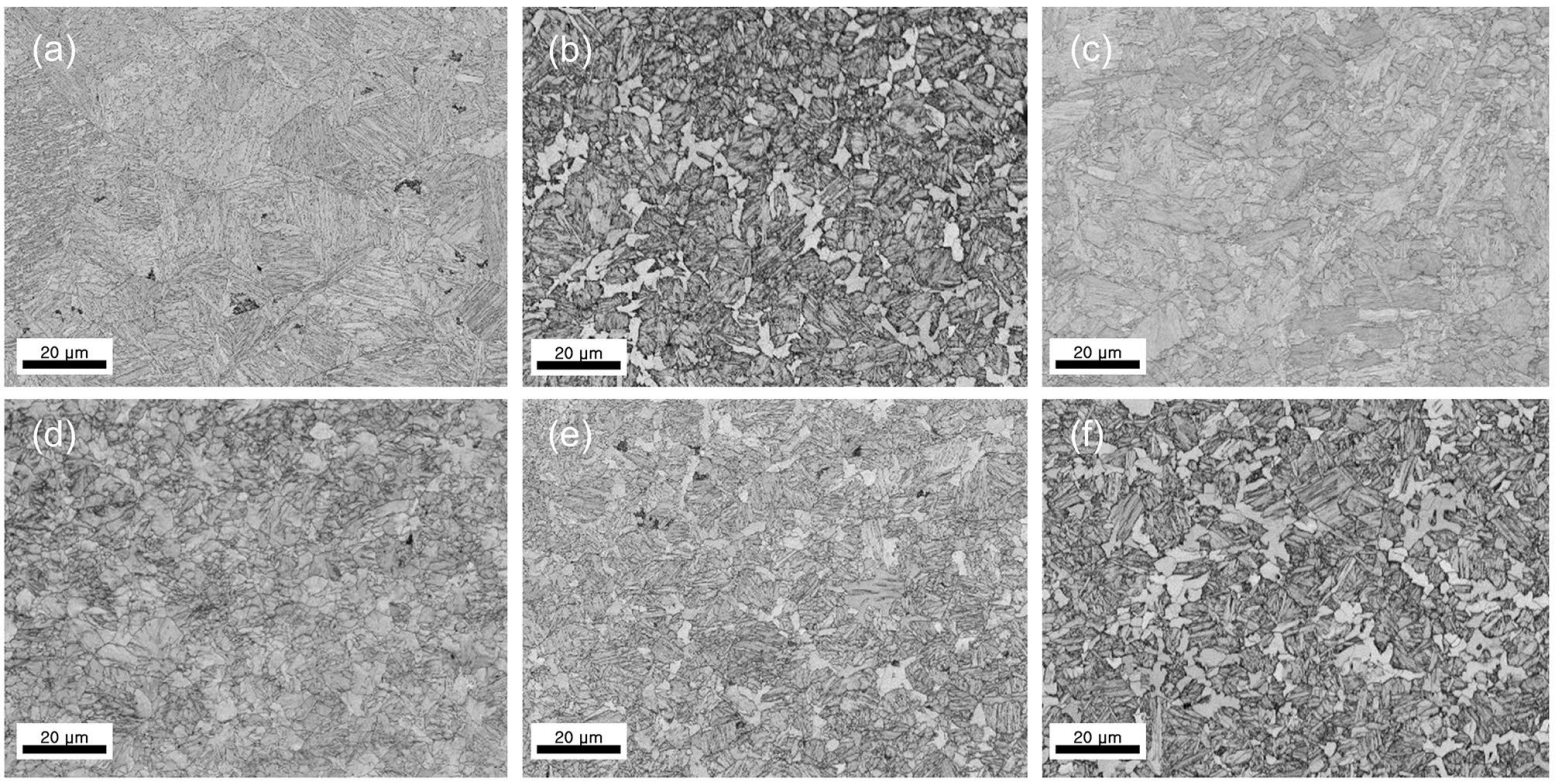
EBSD band contrast (BC) images of different steels. (**a**–**f**) corresponds to Steel A—F in the alphabetic order. Pixel size of images are 1592 by 1196.

In this study, six different types of steels with varying compositions of microstructures were built for both training and testing. Figure 2 shows examples of EBSD images. Table 1 shows the underlying microstructures that make up each type of steel and its proportions while Table 2 shows the chemical composition of each steel.

**Table 1:** Six types of steels with varying compositions of microstructures (all units are in percentage).

	Ferrite	Bainite	Pearlite	Martensite	Remarks
Steel A	0.0	0.0	0.0	100.0	Single phase
Steel B	9.52	0.0	0.0	90.48	Dual phase
Steel C	9.52	90.48	0.0	0.0	Dual phase
Steel D	9.52	35.5	0.0	54.98	Triple phase
Steel E	2.0	4.3	0.1	93.4	Quadruple phase
Steel F	5.7	22.8	0.5	70.9	Quadruple phase

**Table 2:** Chemical composition of each steel (all units are in wt%).

Steel	C	Si	Mn
A, B, C, D	0.24	1.50	1.15
E, F	0.42	0.16	0.60

**Figure 3:**
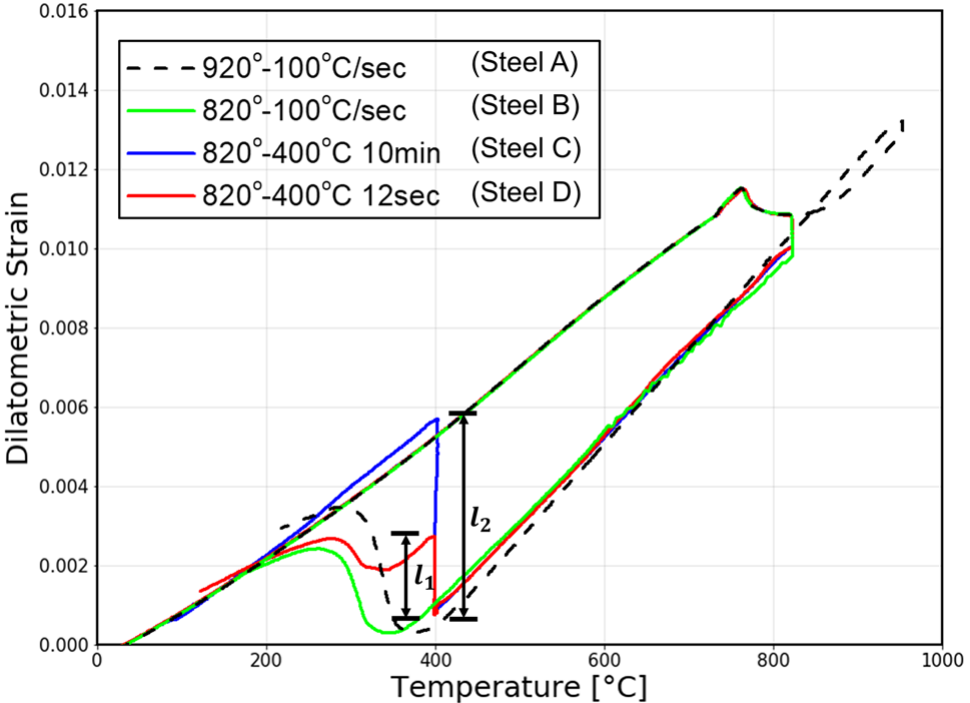
Dilatation curve plot.

As shown in Table 2, the steels can be divided largely into two different sets. The first set of steels (A, B, C, and D) differs from the second set of steels (E and F) in that a different combination of chemical composition was intentionally formed to create a scenario where the sets come from statistically different distributions. Each steel was constructed under different processing conditions. To estimate the true phase fraction of Steel A, B, C, and D, a quench-type dilatometer (Dilatronic III, Theta Inc.) was used and the dilatometric strain is plotted against temperature as shown in Fig. 3. For Steel E and F, JMatPro was used. Before any measurement, a specimen was heated with an induction coil in a vacuum, and a Pt-PtRh (Type R) thermocouple was attached to its surface to measure the temperature and dilatometric data at the same time^18^.

A dilatometer measures the thermal expansion strain of a specimen during a heat cycle. If a phase transformation occurs leading to a change in the thermal coefficient, there will be an inflection in the dilatometric curve, which indicates the start of the transformation. As shown in Fig. 3, Steel A is cooled down to room temperature at the rate of 100$^\circ$C per second. Steel B had the slope of the dilatometer curve unchanged until the transformation to a specific phase when it was cooled down from 820$^\circ$C to room temperature. This indicates it is composed of ferrite and another phase. We could assume that the phase other than ferrite was martensite by calculating the transformation temperatures using the given empirical formula^19^. For calculating the formula, the chemical composition of the austenite phase at transformation is assumed equal to the chemical compositions of the raw material and of the austenite in the two-phase equilibrium since the change in slope of cooling curve before phase transformation is unclear. To be specific, based on the formula, the MS temperatures turned out to be 403$^\circ$C and 369$^\circ$C for Steel A and B respectively. As the measured transformation temperatures (396$^\circ$C and 358$^\circ$C) turned out to be lower than the calculated ones and the transformation was fast (please refer to Figure S2 in Supplementary Information for transformation speed), we decided the phase was martensite. The proportion of the two phases was estimated using SEM images of etched specimens by labeling the percentage of the bright regions as ferrite and the dark regions as martensite. For the case of Steel C, it was cooled down from 820 to 400$^\circ$C and left there for 10 min before it was cooled down again. Since Steel C and D saw no transformation of austenite to ferrite during the cool down above 400$^\circ$C, they were considered to have the same amount of ferrite as Steel B. We knew that bainite was created because the transformations at 400$^\circ$C occurred at a higher temperature than the calculated MS temperature of Steel B, and they happened at a relatively slower pace. To estimate the percentage of bainite in the steels, the ratio of dilatation by bainite transformation during a 12-s hold (${l}_{1}$) to dilatation by the transformation of austenite to bainite at 400$^\circ$C (${l}_{2}$) was multiplied by the percentage of austenite. No residual austenite was observed for any of the aforementioned process conditions. Further details regarding the estimation of phase ratio are provided in Supplementary Information (Figure S1, S2). Process conditions for Steel E and F are listed in Table S1. The dilatation curve plot of Steel E and F is not provided.

### Description of extracted features

**Figure 4:**
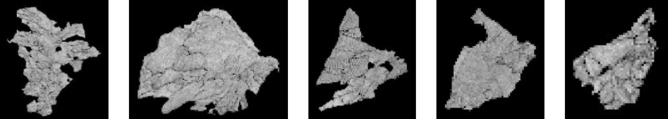
Examples of segmented EBSD images by grain (Steel D). Pixel size of the leftmost image is 90 by 86.

By using TIMS software developed by Korea Institute of Materials Science, we analyzed and extracted 19 features from each segmented EBSD image shown in Fig. 4. It should be noted that the images of different pixel sizes were segmented using the software based on grain boundary. The description of the features is summarized in Table 3. Some of the features include grain size (diameter), grain average misorientation (GAM), the standard deviation of KAM, solidity, area-weighted average sharpness, etc. They are commonly considered to be the main contributive factors to distinguish different microstructural classes in many works of literature^1,4,6,8,11^, though there exists no rule-of-thumb for which one is more important than another in distinctive cases. For an intuitive understanding of some of the features, further details are provided in Supplementary Information (Table S2). The training data consists of 116,811 samples without any labels. Although the true phase fraction of each steel is known in advance from observing the dilatometer curve, labels for each grain on the EBSD images are left to be discovered by the proposed method in this study. 20% of raw data was used as a test set.

**Table 3:** Description of input features.

	Input feature	Symbol and description
1	Grain size	${d}_{s}=2\times \sqrt{\frac{p\times {s}^{2}}{\pi }}$
2	GAM	$\mathrm{GAM}=\frac{{\sum }_{i}{KAM}_{i}}{p}$
3	Std. of KAM	${\sigma }_{KAM}, {KAM}_{(i,j)}=\frac{\sum {g}_{(i,j)}{g}_{(i+x,j+y)}^{-1}}{k}$
4	GOS	$\mathrm{GOS}=\frac{{\sum }_{i}{g}_{ave}{g}_{i}^{-1}}{p}$
5	Solidity	$S=\frac{{A}_{i}}{{A}_{c}}$
6	Avg. of misorientations at the boundary	${\mu }_{mis}$
7	Aspect ratio	$AR=\frac{w}{h}$
8	Weighted avg. sharpness	${\mu }_{sharp}=\frac{{\sum }_{i}{w}_{i}\times {sharpness}_{i}}{{\sum }_{i}{w}_{i}}, sharpness=\frac{D}{d}$
9	Avg. band contrast in the grain	${\mu }_{BC}=\frac{{I}_{max}-{I}_{min}}{{I}_{max}-{I}_{min}}$
10	Std. of band contrast within the grain	${\sigma }_{BC}$
11	Area weighted avg. grain size of neighbor grains surrounding the current grain	${\mu }_{GS}$
12	Area weighted avg. GAM of neighbor grains surrounding the current grain	${\mu }_{GAM}$
13	Area weighted avg. std. of GAM of neighbor grains surrounding the current grain	${\mu }_{\sigma GAM}$
14	Area weighted avg. GOS of neighbor grains surrounding	${\mu }_{GOS}$
15	Area weighted avg. aspect ratio of neighbor grains surrounding	${\mu }_{AR}$
16	Area weighted avg. band contrast of neighbor grains surrounding	${\mu }_{nBC}$
17	Area weighted avg. std. of band contrast of neighbor grains surrounding	${\mu }_{\sigma nBC}$
18	Area weighted avg. solidity of neighbor grains surrounding	${\mu }_{S}$
19	Area weighted avg. sharpness of neighbor grains surrounding	${\mu }_{nS}$

$p$ is the number of pixels in a grain. $s$ is the step size.$g$ is user-defined kernel and $k$ is the number of kernel elements. ${A}_{i}$ denotes the area of an image while ${A}_{c}$ is the area of convex hull image.$w$ and $h$ are weight and height of grain respectively. ${w}_{i}=d\times D/2$, where $d$ and $D$ are distance and convexity depth. $I$ represents luminance.

### Implementation of deep learning

For the implementation of deep learning, we used Python 3.5.2 and Keras 2.2.5. For training, a GPU (RTX 2080) was used for fast computation. In this study, 1D InfoGAN was implemented because the type of training data is numerical (a single row in the table corresponds to 19 extracted feature values for one segmented image). Training a GAN model is often difficult due to the stochasticity of its nature. For training images, the traditional method is to output the generated sample image, compute the evaluation scores^20^, and save the current model for every pre-defined epochs. This allows the user to check if the generated sample is realistic (to the eyes of the beholder) and use the saved GAN model of the same epoch. Training a 1D GAN is particularly challenging in some aspects because, unlike images, there exists no established method to visualize high-dimensional data to effectively monitor the ongoing training status of the GAN model. Therefore, an alternative is to monitor an appropriate evaluation score and visualize as many data distributions as possible. Here, L2 reconstruction error was used as the evaluation measure, and four different combinations of 2D graphs were plotted to monitor the data points being generated. The reconstruction error is equivalent to the mean squared error between the training samples and generated samples. It was computed for every 100 epochs and displayed while playing the minimax game, as stated in Eq. (5). Given a generator $G$ and a set of training data $X=\left\{{x}_{1}, {x}_{2}, {x}_{3}, \dots , {x}_{n}\right\}$, the reconstruction error is defined by:

6 $${\mathcal{L}}_{rec} \left( {G,{ }X} \right) = \frac{1}{n}\mathop \sum \limits_{i = 1}^{n} \left| {\left| {G\left( {z, c} \right) - x_{i} } \right|} \right|^{2}$$

**Figure 5:**
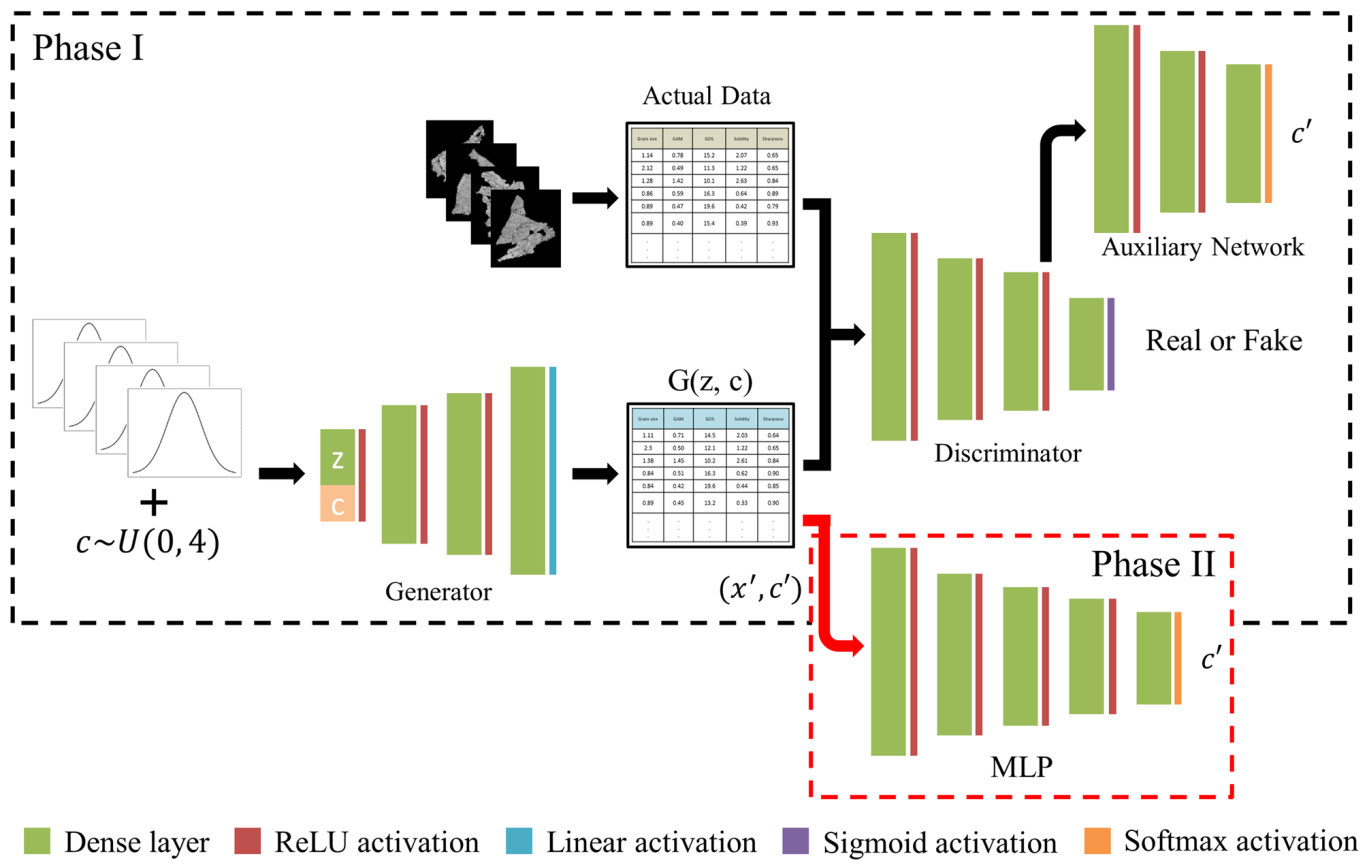
InfoGAN and MLP classifier structures are shown inside dashed black and red boxes, respectively. In Phase I, InfoGAN is trained. In Phase II, the generator creates samples and labels $({x}^{^{\prime}}, {c}^{^{\prime}})$ that are given as input to MLP for training. Then, the trained model performs the classification of raw data.

As mentioned in “Revision of recent generative deep learning techniques”, the structure of InfoGAN is similar to that of an ordinary GAN except for a few extensions due to the latent code $c$. Figure 5 illustrates the structure of InfoGAN and that of an MLP of which the training procedure is indicated as a black and a red arrow, respectively. They are also presented as Phase I and II, respectively because they are two separate models and are not trained simultaneously by sharing a loss function. Phase II comes after Phase I. To elaborate, after Phase I (training InfoGAN) is over, what is created by the generator with labels is given as input to the MLP for training (Phase II). In Phase I, a Gaussian noise matrix $z$ concatenated with random latent code $c$ is given as input to the generator, hence the representation $G\left(z, c\right)$ for the generator output. The generator is an MLP with an input layer, two hidden layers and an output layer the size of the total feature counts. Similarly, the discriminator is an MLP with an input layer, two hidden layers, but with two output layers, one for the discrimination and the other for the control variable or the latent code ${c}^{^{\prime}}$. For each output layer, binary cross-entropy and categorical cross-entropy were adopted respectively for computing loss. Inputs to the discriminator are both raw data $x$ and generated sample $G\left(z, c\right)$.

For training the aforementioned InfoGAN model, He initializer was used to initialize the weights in the hidden layers. Implementing Adam optimizer, the learning rate was set to 2e−4 while the exponential decay set to 0.5. Hyper-parameters, including the number of epochs and batch size were set to 30,000 and 512, respectively. The dimension of the latent code depends on the prior knowledge of the number of classes of raw data. Training the generator and the discriminator was practiced one at a time, meaning that while updating the weights of the generator, the discriminator was not trained and vice versa. The proposed architecture of the InfoGAN for the case of Steel E is summarized in Table 4. Since Steel E is an example of a quadruple phase, the latent code with a dimension of four is appended to the latent space $z$, which was heuristically determined to have the same dimension. ReLU activation function is applied to the networks after every dense layer except for the output layers to account for the nonlinearity in the training data. However, different activation functions are utilized after the output layers depending on the purposes of each network. For example, the auxiliary network has a softmax activation function because it is required to output multiple labels.

**Table 4:** The proposed InfoGAN structure.

Generator network ($G$)			Discriminator network ($D$)			Auxiliary network ($Q$)		
Layer	Type	Dimension	Layer	Type	Dimension	Layer	Type	Dimension
Input	Latent ($z$) + Code ($c$)	4 + 4	Input	Feature	19	Input	Hidden layer	50
Hidden 1	Dense layer	50	Hidden 1	Dense layer	100	Hidden 1	Dense layer	50
	ReLU activation	–		ReLU activation	–		ReLU activation	–
Hidden 2	Dense layer	50	Hidden 2	Dense layer	50	Hidden 2	Dense layer	20
	ReLU activation	–		ReLU activation	–		ReLU activation	–
Output	Dense layer	19	Output	Dense layer	1	Output	Dense layer	4
	Linear activation	–		Sigmoid activation			Softmax activation	–

Each network holds two hidden layers but with a varying number of nodes. Input to the auxiliary network is the output of the second hidden layer in the discriminator.

When the training of InfoGAN is completed, the generator is capable of sampling points from learned data distributions of a specified control variable. For instance, since Steel C had been known before to be dual-phase, it was trained to have two distinct data distributions from which the generator later sampled points. Then, the generated samples $({x}^{^{\prime}}, {c}^{^{\prime}})$ are provided as input to an MLP classifier, as shown in Phase II. After scaling the data from 0 to 1, the dataset was split into a train and a test set at a ratio of 8–2. The MLP classifier consists of an input layer of 19 dimensions, three hidden layers, and an output layer with the size depending on the number of classes of the steel in concern. For all steels, the test accuracy turned out above 99%, implying the models have been optimized. Last but not least, the raw data which had been used to train InfoGAN is put through the optimized feed-forward classifier network to give out the labels. The estimated phase fraction of each steel is presented in “Sec10”.

## Results and discussion

### Estimation of phase volume fraction

The result summarized in Table 5 implies the high feasibility of using the proposed method for a fast phase fraction estimation for steels or even any other materials without labeling all the training data. Though none of the estimated values is the same, all of them are on the right track as the models can distinguish the big and small chunks. The mean relative error for the estimations implies the high feasibility of using the proposed method. It shows that the mean relative error can reach at most 4.53% while it can be as low as 0.73%, which is very close to the exact estimation. Furthermore, the result implies the proposed method is suitable for steels with different kinds of chemical compositions (Steel A, B, C, D, and Steel E, F). It is also inspirational in the sense that no domain knowledge of steel processing is necessary to estimate the phase fractions.

**Table 5:** True and estimated phase fraction of each type of steel (all units are in percentage).

	Ferrite	Bainite	Pearlite	Martensite	Relative ERROR
**Steel A**					
True PF	0.0	0.0	0.0	100.0	1.87
Estimated PF	2.1	0.0	0.7	97.2	
**Steel B**					
True PF	9.5	0.0	0.0	90.5	3.18
Estimated PF	2.7	0.0	0.7	96.6	
**Steel C**					
True PF	9.5	90.5	0.0	0.0	2.2
Estimated PF	11.7	88.3	0.0	0.0	
**Steel D**					
True PF	9.5	35.5	0.0	55.0	4.53
Estimated PF	10.4	28.7	0.0	60.9	
**Steel E**					
True PF	2.0	4.3	0.1	93.4	0.73
Estimated PF	2.0	3.4	0.0	94.6	
**Steel F**					
True PF	5.7	22.8	0.5	70.9	3.1
Estimated PF	5.5	17.2	0.2	77.2	

**Figure 6:**
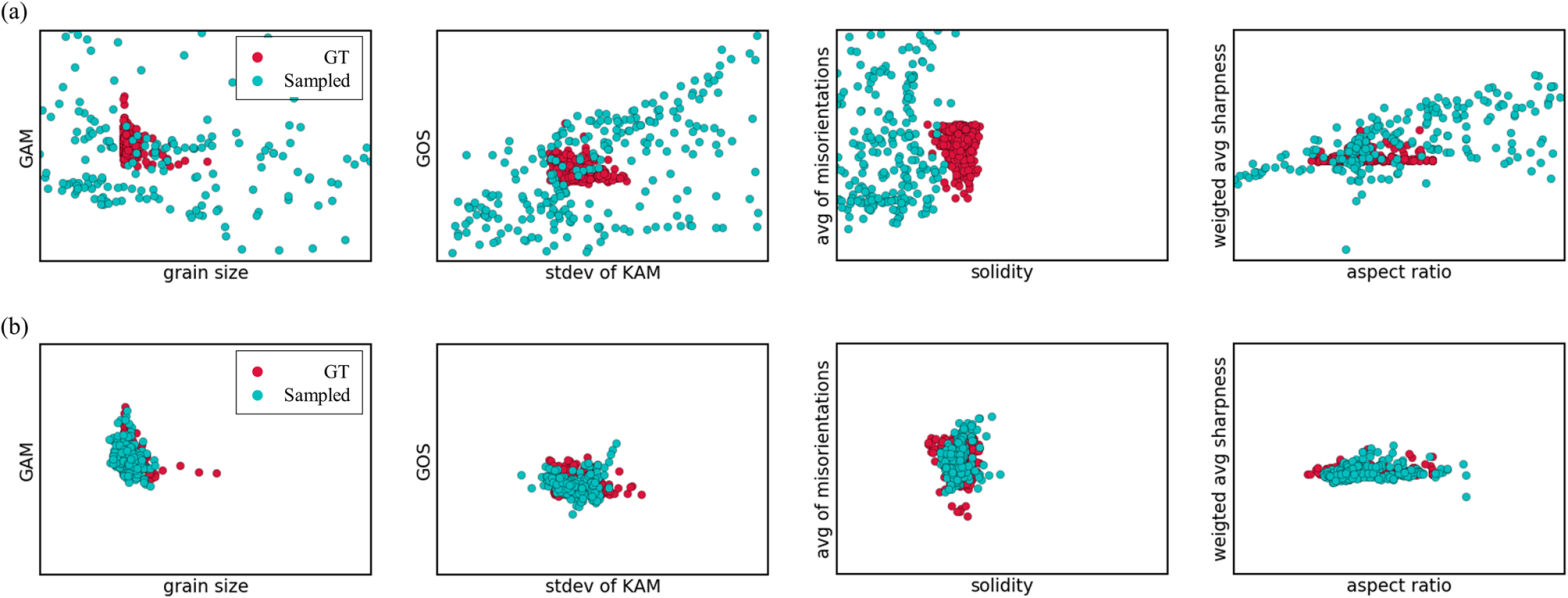
(**a**) Sampled points at epoch 100 for Steel E. (**b**) Sampled points at epoch 11,000 for the same steel. The ground truth is shown in red dots while the sampled points are in green dots.

Figure 6 shows an intuitive form of visualizing the data distributions, which demonstrates the gradual change in the form of distributions as optimization proceeds. The optimized InfoGAN models were stored during training by screening both the L2 reconstruction error and the data distributions. Here, the red dots are the ground truths for Steel E, while the green dots are the sampled points at the specified epochs. It can be seen from (a) and (b) that as the model gets optimized, sampled points get closer to the ground truth meaning that they eventually come from more similar underlying data distributions. Not all results are shown for clarity.

Table 6 compares the relative errors of the proposed approach with a number of baseline clustering models, showing that it outperforms them for the estimation of phase volume fraction in all steels. These models are composed of pure clustering models such as k-means, Gaussian mixture model (GMM), and density-based spatial clustering of applications with noise (DBSCAN) and combinations of various dimension reduction techniques and DBSCAN. For the combinations, the dimension reduction techniques are principal component analysis (PCA), autoencoder, and GAN that are denoted as P-, A-, and G- respectively. DBSCAN was selected as the sole clustering model after dimension reduction because of its superior result compared to the other two models. This implies that the proposed method is so far the most preferable choice for the phase classification of steels in an unsupervised manner.

**Table 6:** Comparison of the proposed approach with baseline clustering models. Best results presented in bold font.

	Steel A	Steel B	Steel C	Steel D	Steel E	Steel F	Mean (%)
**Relative error (%)**							
K-means	4.8	35.8	6.8	14.8	28.5	20.4	18.5
GMM	5.2	21.4	6.8	6.5	24.1	22.0	14.3
DBSCAN	2.8	8.5	8.3	6.8	19.6	3.1	8.2
P-DBSCAN	2.8	6.2	6.8	6.4	10.6	5.5	6.3
A-DBSCAN	1.9	8.7	4.3	8.7	1.3	**3.0**	4.7
G-DBSCAN	1.9	8.8	5.7	8.0	1.9	3.1	4.9
InfoGAN	**1.9**	**3.2**	**2.2**	**4.5**	**0.7**	3.1	**2.6**

### Evaluation of the fraction estimation

For evaluation, the probability density function was plotted at four different epoch points to monitor if the learned data distribution was getting close to the ground truth as the training progressed. The probability density function (PDF) was approximated by kernel density estimation (KDE) or Parzen-Rosenblatt window method, which is a non-parametric way to estimate the PDF of a random variable^21^. In statistics, it makes an inference about the population based on a finite data sample by applying Gaussian kernels with a pre-defined bandwidth. Suppose $({x}_{1}, {x}_{2}, \dots , {x}_{n})$ are i.i.d. samples drawn from a distribution with an unknown density $f$. The kernel density estimator is defined as follows:

7 $$\hat{f}_{h} \left( {\text{x}} \right) = \frac{1}{n}\mathop \sum \limits_{i = 1}^{n} K_{h} \left( {x - x_{i} } \right) = \frac{1}{nh}\mathop \sum \limits_{i = 1}^{n} K\left( {\frac{{x - x_{i} }}{h}} \right)$$

In Eq. (7), $K$ represents a kernel, and $h$ is a smoothing parameter or the bandwidth. Its intuitive meaning is that normal kernels on each of the data points are summed to make the final kernel density estimate. For the computation, a built-in Pandas module was used, and we set the smoothing parameter $h$ to 0.6.

**Figure 7:**
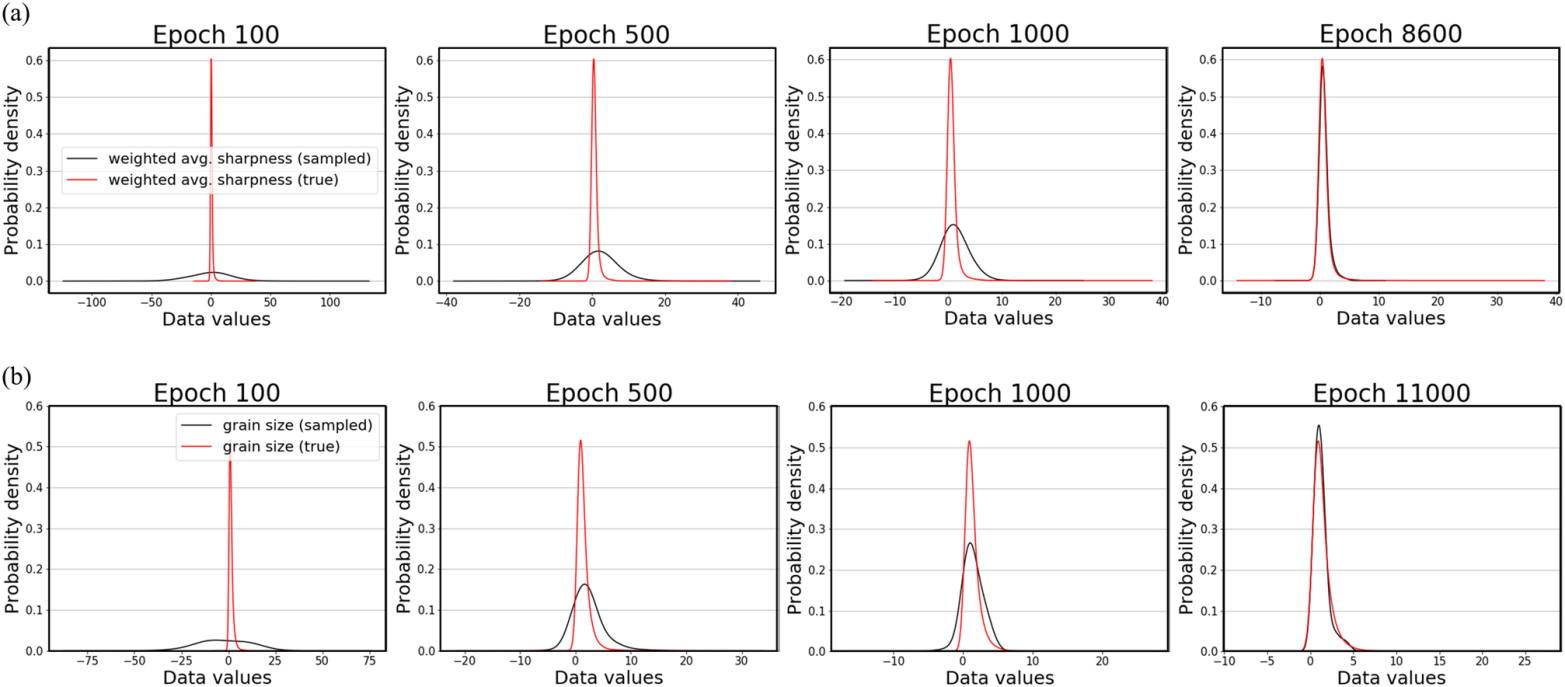
(**a**) PDFs for Steel E. (**b**) PDFs for Steel F. The ground truth is shown in a red curve while the estimated PDF is in a black curve.

Figure 7 shows the plotted PDFs at four different epoch points for Steel E and F. Looking at the plots, the red line represents the ground truth PDF, whereas the black line represents the estimated PDF. As the epoch number increases, the estimated PDF becomes similar to the ground truth. At the final epochs, the PDFs almost overlap, indicating that much similar data distributions have been learned by the models.

**Figure 8:**
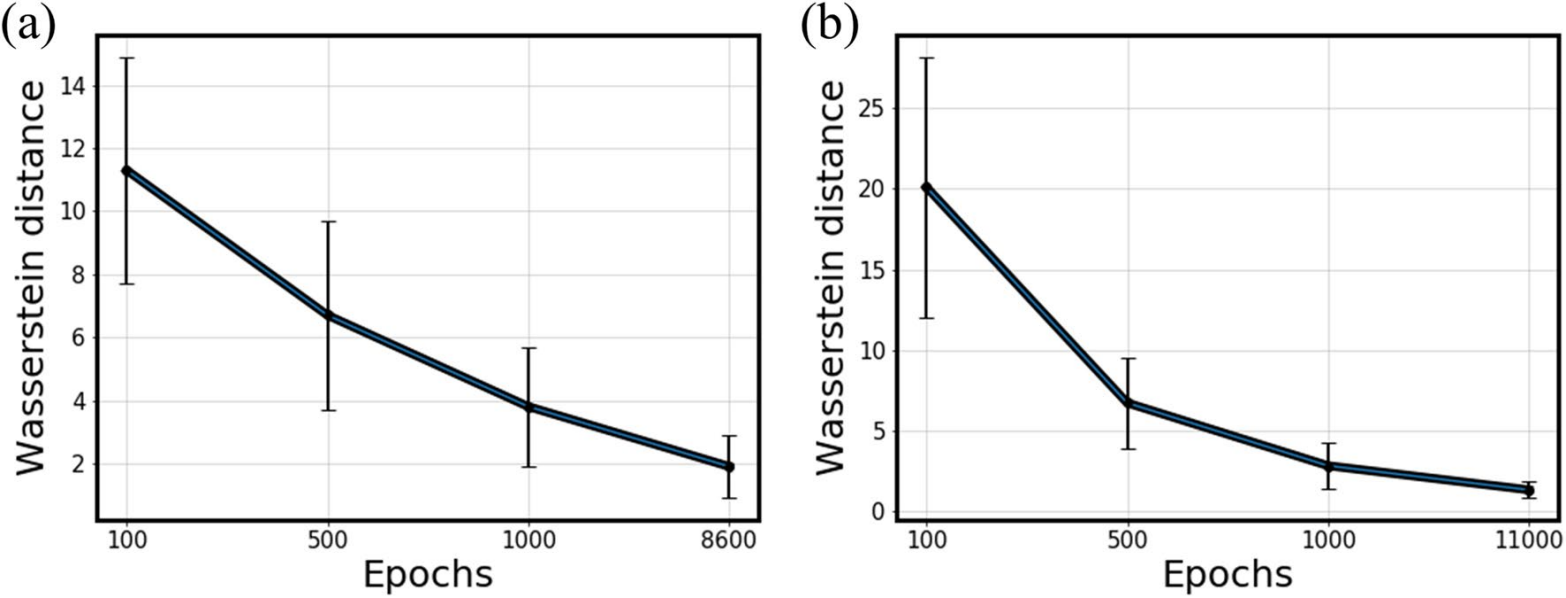
Average Wasserstein Distances at different epochs. (**a**) Steel E. (**b**) Steel F.

Besides, Wasserstein Distance (WD)^22^ was measured to quantify how similar two probability distributions are. Figure 7 demonstrates how similar the learned distributions are to the ground truths for only a few features. It is necessary to evaluate the overall similarity for all features combined. Averaging out WDs for all features solves this problem. Figure 8 presents the gradual decrease in average WDs for Steel E and F as training progressed. It can be concluded that the data distributions were learned similarly for all features, not just for the few ones. Table 7 summarizes it for the rest of the steels. In the table, ‘Best’ indicates the epoch point for which each model had the best performance (the least reconstruction error).

**Table 7:** Summary of average Wasserstein distances at different epochs.

	Epochs			
	100	500	1000	Best
**Average Wasserstein distance**				
Steel A	14.15 $\pm$ 4.27	6.25 $\pm$ 2.86	3.57 $\pm$ 1.54	1.48 $\pm$ 0.47
Steel B	16.82 $\pm$ 6.31	3.21 $\pm$ 1.26	2.38 $\pm$ 1.19	1.16 $\pm$ 0.41
Steel C	7.79 $\pm$ 1.87	1.52 $\pm$ 0.47	0.89 $\pm$ 0.29	0.76 $\pm$ 0.19
Steel D	8.88 $\pm$ 2.20	2.20 $\pm$ 0.97	1.62 $\pm$ 0.82	0.77 $\pm$ 0.22
Steel E	11.28 $\pm$ 3.65	6.74 $\pm$ 3.00	3.81 $\pm$ 1.85	1.91 $\pm$ 1.01
Steel F	20.14 $\pm$ 8.08	6.65 $\pm$ 2.81	2.84 $\pm$ 1.37	1.27 $\pm$ 0.53

**Figure 9:**
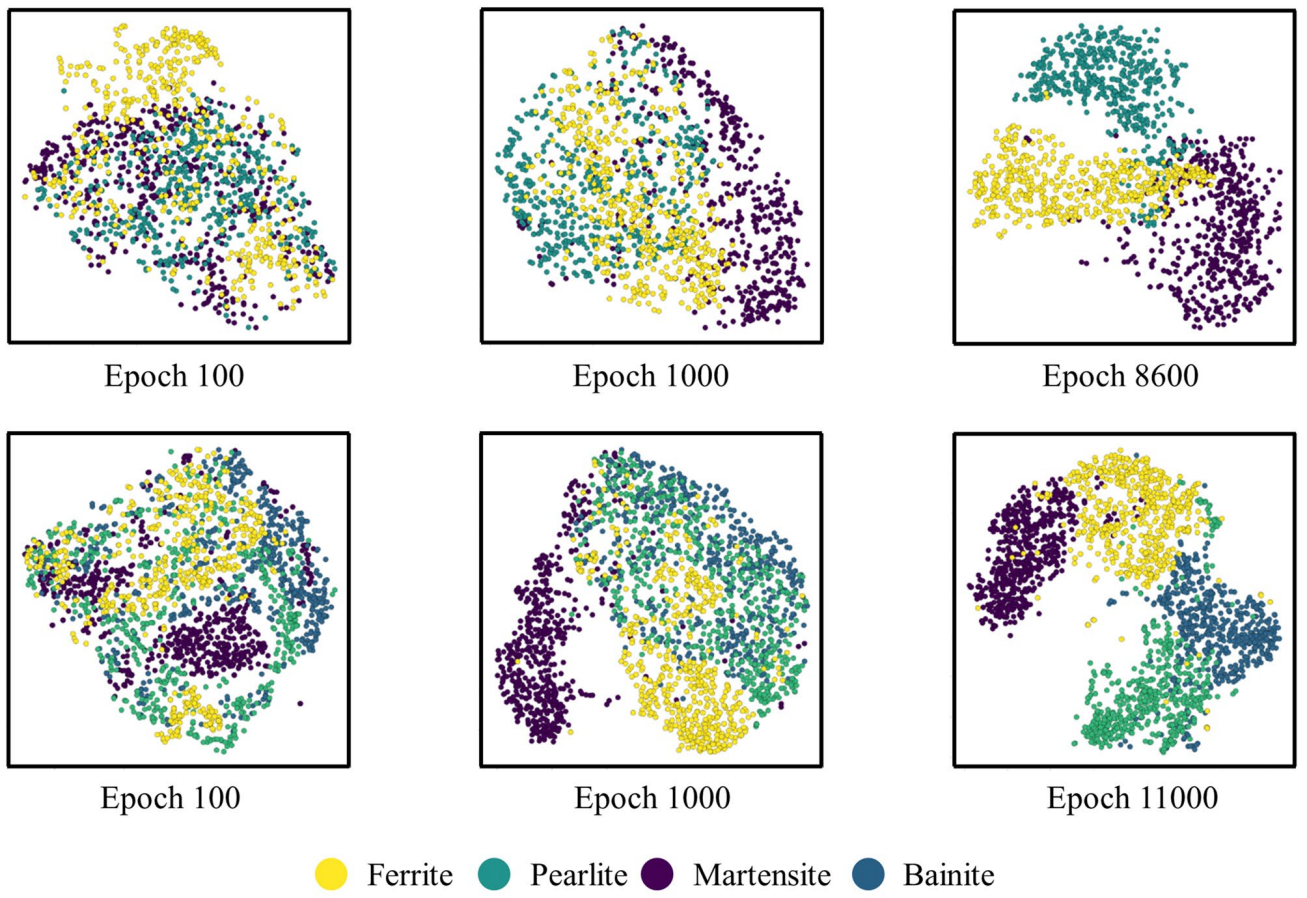
t-SNE visualization of generated features at different epochs. The top row represents the embedded feature distributions for Steel E while the bottom row does it for Steel F.

Figure 9 shows the feature visualizations using t-SNE^23–26^ on the InfoGAN generated features for Steel E and Steel F. t-SNE is a widely used technique for visualizing high-dimensional data into 2D or 3D by projecting data into a low dimensional space so that the clustering in the high-dimensional space is preserved. It measures the similarity on a t-distribution of each point in data based on the distance of points and clusters them based on the similarity scores. For its iterative optimization, the Kullback Leibler divergence of the distributions in both high-dimension and low-dimension is minimized. In Fig. 9, the top row represents the embedded feature distributions for Steel E while the bottom row does it for Steel F. The noteworthy part of it is that as the optimization gradually reaches the minimum, the features representing each phase become distinctively separable from one another. This implies the fact that at the optimal point, the model is capable of distinguishing the unique characteristics of each feature and thus create similar points from the learned distributions.

### Feature importance of input features

In many engineering fields, it is often extremely helpful to know which features turned out to be critical for making the phase classification. Highlighting the input features a deep learning model uses to support its prediction has been an essential part of explainable artificial intelligence^27–30^. Though several techniques^31–33^ have been introduced to unveil the underlying mechanism of a deep neural network, one of the most promising ones nowadays is the Layer-wise Relevance Propagation (LRP)^34^ which we adopted in this study. When computing the amount of contribution that each input makes to the output of a neural network, it can be denoted as the partial derivative of the output with respect to the input.

8 $$f\left(x\right)=f\left(a\right)+\sum_{p=1}^{d}\frac{\partial f}{\partial {x}_{p}}{|}_{x=a}\left(x-a\right)+\epsilon$$

**Figure 10:**
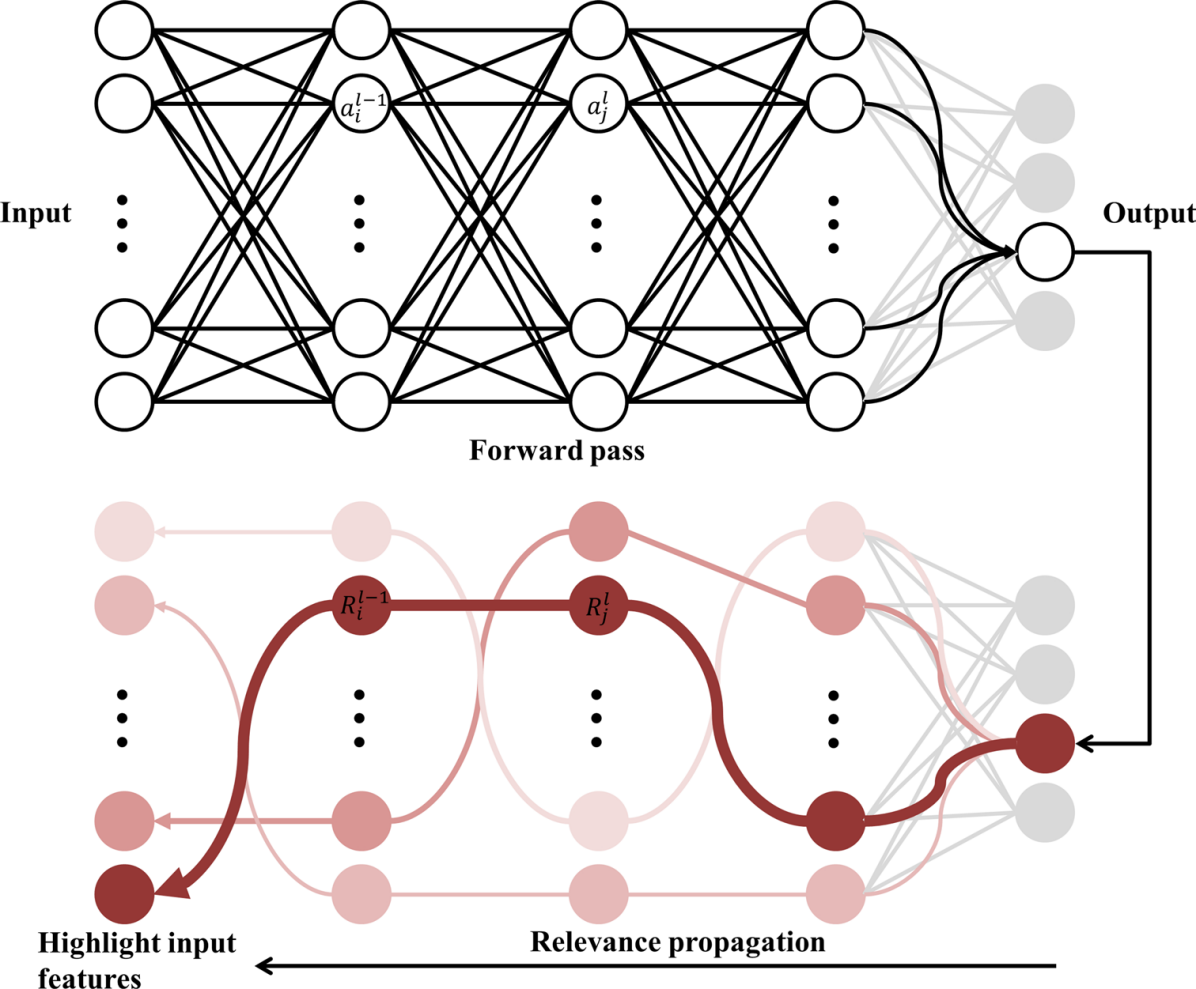
Schematic diagram showing the general process of LRP. Starting from the output from a trained MLP, the relevance score is distributed backward. The input feature from the leftmost layer that is assigned the highest relevance score is colored in dark red.

As shown above, the second term of the Taylor series^35^ in its first-order with the higher-order terms represented as $\upepsilon$ can be understood as describing the change in the output $f(x)$ as ${x}_{p}$ varies. For the LRP technique, an appropriate ‘$a$’ is searched so that the first and the higher-order terms erases out, leaving $f(x)=\sum_{i=1}^{d}{R}_{i}$ where ${R}_{i}$ is the relevance score interpreted as the contribution score. The formula can be reformulated as follows for any given two consecutive layers in a deep neural network: $\sum_{i}{R}_{i}=\sum_{j}{R}_{j}$. This is equivalent to a conservation property, where what has been received by a neuron must be redistributed to the lower layer in an equal amount^34^. Figure 10 illustrates the general process of the LRP.

In LRP, the prediction output of a neural network propagates backward based on the trained weights and biases, and the relevance score is calculated by Eq. (9):

9 $${R}_{i}^{(l-1)}={\sum }_{j}\frac{{a}_{i}^{(l-1)}{w}_{ij}^{(l)}}{{\sum }_{i}{a}_{i}^{(l-1)}{w}_{ij}^{(l)}}{R}_{j}^{(l)}$$

${R}_{i}^{(l-1)}$ is the relevance score of the *i*th node in (*l* − 1$\mathrm{th}$) layer. ${a}_{i}^{(l-1)}$ is the activation value while ${w}_{ij}^{(l)}$ denotes the trained weights from the MLP. The weighted sum in the denominator is to ensure that the conservation property holds.

**Figure 11:**
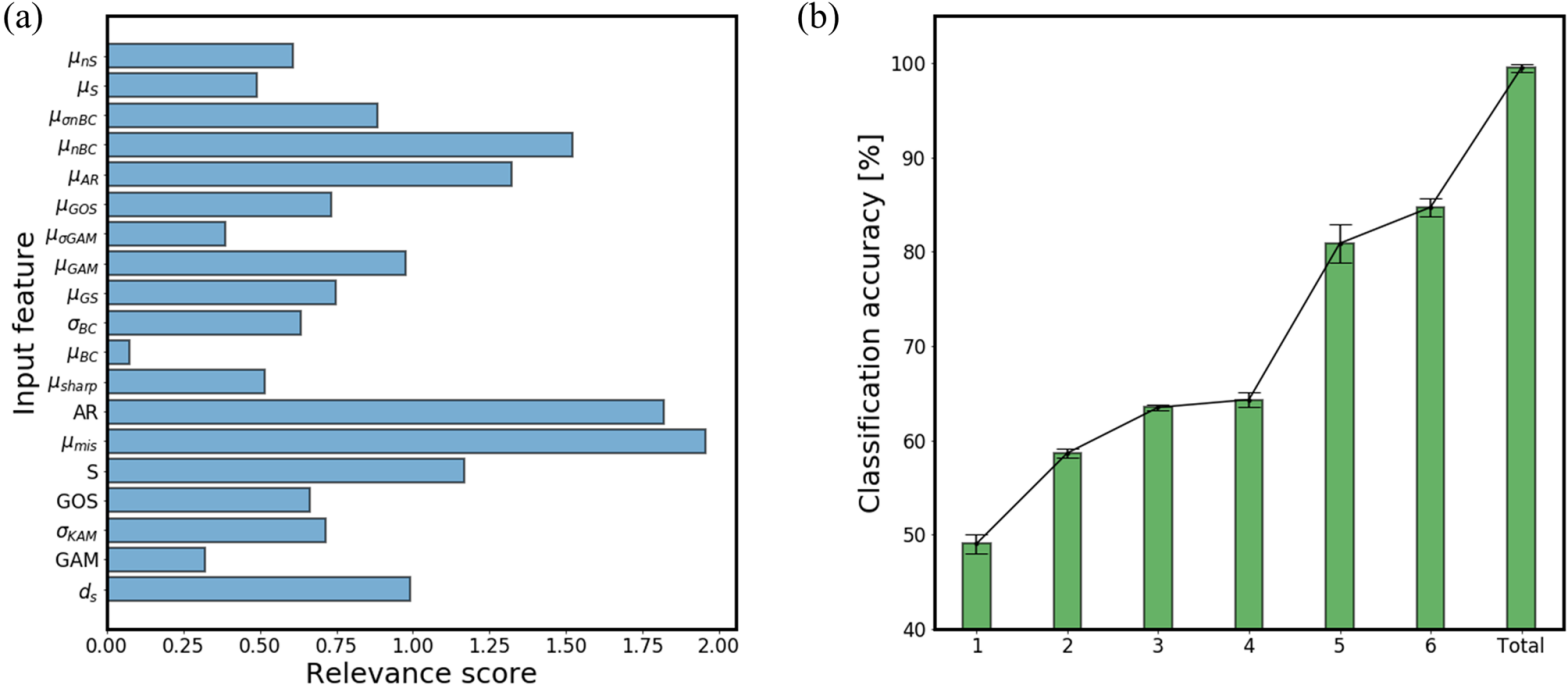
(**a**) Relevance score bar chart of input features of Steel D. (**b**) Comparison of scenarios where ‘Total’ keeps the entire features while the numbers (1–6) in the x-axis represent the number of high-scoring features utilized for phase classification.

In this study, the significance of input features is investigated for Steel D that constitutes the most evenly distributed phase ratios. Of the three phases that constitute the steel, we focus on the classification of martensite that has the largest ratio. Since we present the feature importance using only the LRP technique, we hereby acknowledge the lack of evidence for the following result given solely by the data-driven method, and would like to suggest the important design parameters for further study. Figure 11 (a) is a bar chart that shows the relevance scores assigned to each input feature. The top three high scoring features are ${\mu }_{mis}$, $\mathrm{AR}$, and ${\mu }_{nBC}$ in the sequential order, which is the average of misorientations at the boundary, aspect ratio, and the area-weighted average band contrast of neighbor surrounding grains, respectively. It can be inferred from the result that they should be considered as a priority when designing the composition of the micro-constituents in steel. We verify the result by comparing various scenarios where a different number of high-scoring features are utilized to make phase classification. Figure 11b shows how the elimination of a different set of input features affects the general classification performance of MLP. Keeping the entire features provided the best classification accuracy of 99.5% on average. Whereas using the six top features for training resulted in only a 14.8% reduction in accuracy, using the single top feature had a much larger drop of 50.4%. This implies what has been labeled as more contributive by the LRP technique is in fact, more important in the decision-making.

## Conclusion

In this study, the estimation of phase volume fraction of multi-phase steel via unsupervised deep learning is presented. To the best of our knowledge, it is the first time to solve the problem via unsupervised deep learning, no longer requiring the tedious job of labeling data. The proposed method suggests a generalized approach to identify and quantify the classes of multi-phase steel, revealing the possibility for even non-experts without any prior knowledge to do the task. In total, six different types of steel with varying microstructure compositions were tested. The result shows the estimated phase fractions to be a good match with the true phase fractions for all tests. Furthermore, it implies the proposed method is suitable for steels with different kinds of chemical compositions (Steel A, B, C, D, and Steel E, F). This is made possible by implicitly learning the data distribution similar to that of the training data by using a type of a generative model, InfoGAN. An optimized generator is then able to output a paired dataset controlled by the latent code specified by the user. Next, an MLP classifier is trained using the generated dataset and performs a prediction on the raw data to provide them with labels. Several visualization techniques including t-SNE were implemented to validate the aptness of the utilized InfoGAN model. Lastly, the significance of input features is assessed by using LRP. We hope that this work can contribute to making a leap forward in the automation of phase quantification that has previously been a laborious task. We also believe the proposed method can be widely incorporated in the industries as well as the laboratories for research purposes.

## Supplementary Information

Supplementary Information

## Data Availability

The datasets generated during and/or analyzed during the current study are available from the corresponding author on reasonable request.
