# Supplementary material for: Estimating the phase volume fraction of multi-phase steel via unsupervised deep learning
Source: Sci Rep. 2021 Mar 15;11:5902. doi: 10.1038/s41598-021-85407-y (PMC7971040; doi:10.1038/s41598-021-85407-y)
Supplement: Supplementary file 1 — Supplementary Information [file 41598_2021_85407_MOESM1_ESM.pdf]

Supplementary Information

ESTIMATING THE PHASE VOLUME FRACTION OF MULTI-PHASE STEEL VIA UNSUPERVISED DEEP LEARNING

Sung Wook Kim[1], Seong-Hoon Kang[2], Se-Jong Kim[2*], and Seungchul Lee[1,3,4*]

[1]Department of Mechanical Engineering, Pohang University of Science and Technology, 77 Cheongam-ro, Pohang, Republic of Korea

[2]Korea Institute of Materials Science, 797 Changwon-daero, Seongsan-gu, Changwon, Republic of Korea

[3]Graduate School of Artificial Intelligence, Pohang University of Science and Technology, 77 Cheongam-ro, Pohang, Republic of Korea

[4]Institute of Convergence Research and Education in Advanced Technology, Yonsei University, 50 Yonsei-ro, Seoul, Republic of Korea

* Two corresponding authors

Email address:

kswltd@postech.ac.kr (Sung Wook Kim)

kangsh@kims.re.kr (Seong-Hoon Kang)

ksj1009@kims.re.kr (Se-Jong Kim)

seunglee@postech.ac.kr (Seungchul Lee)

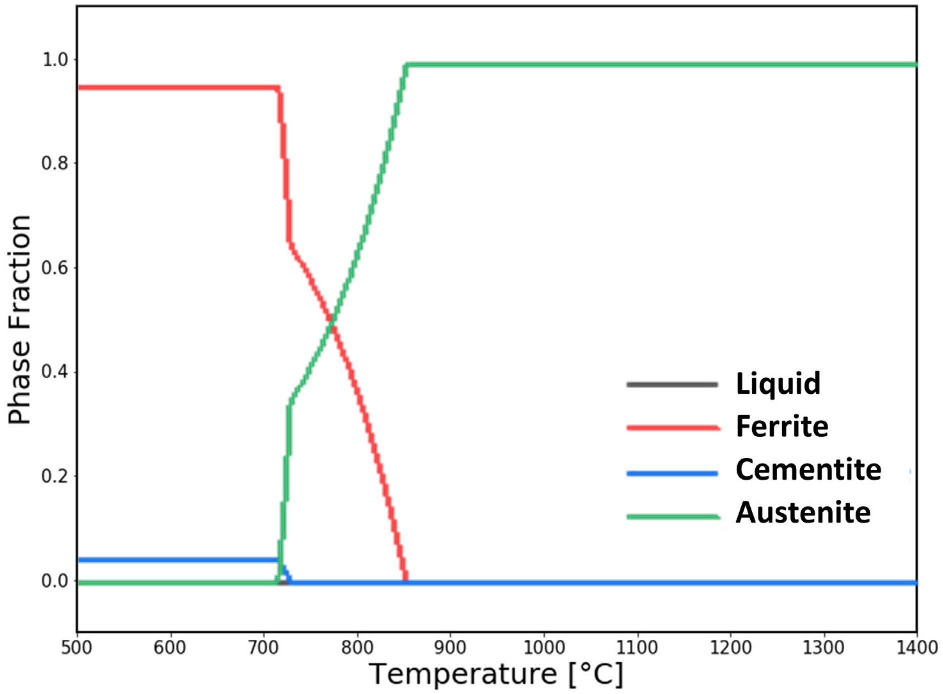

Figure S1. Calculated phase fraction curve with respect to temperature. Between the red and green lines (710°C - 890°C) is interval in which ferrite and austenite coexist. In Figure 3, the black dashed line grows steadily until 710 ° C where it starts to decrease forming a curve. In case of Steel A, since only one transformation occurs afterwards and no residual austenite was found, it can be assumed that the original phase all turned into martensite. The transformed phase is martensite because the start of the transformation temperature is slightly lower than the output of the empirical formula given the observed carbon concentration, and the transformation happened relatively fast as shown in Figure S2. For Steel B, there is originally ferrite only followed by the occurrence of another phase, which in this case is martensite for the same reason. Thermal dynamic calculation was carried out using Thermalcalc software based on TCFE 9.0 database.

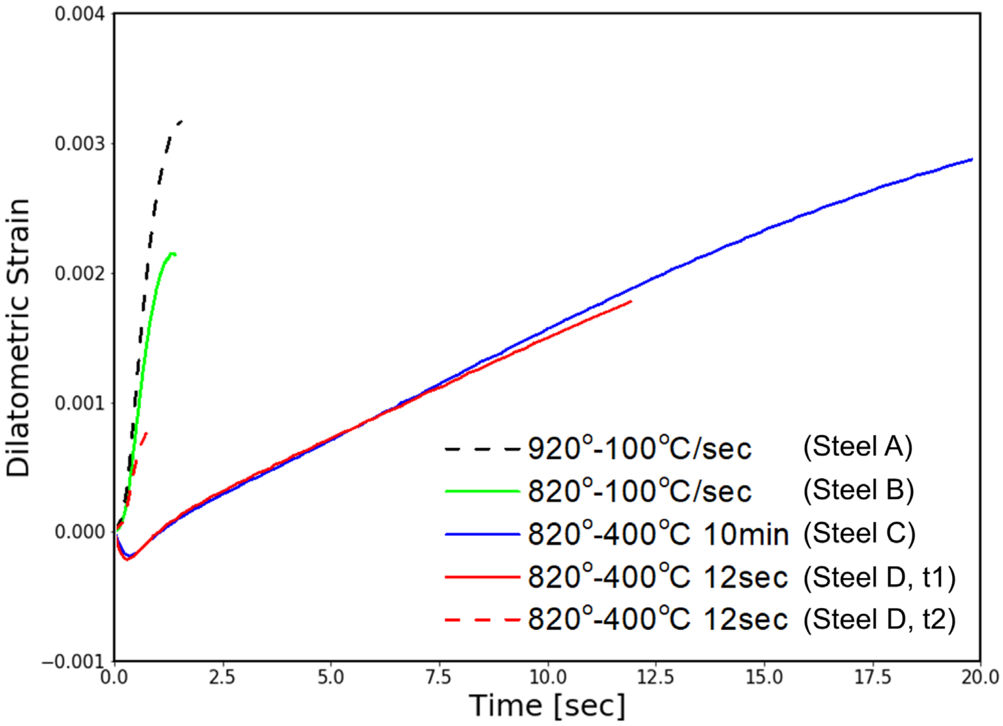

Figure S2. Dilatometric strain curve with respect to time during phase transformation used to analyze the occurence of bainite, martensite, or both in steels. In case of Steel C, the blue line gets flat after 110 seconds is past (not shown in the graph) meaning no change in dilatation strain is observed. This indicates there is no more phase transformation. For Steel D, two transformations happened under 400°C. The first one (red line, t1) shows transformation speed that is not different from that of Steel C. Therefore, the transformed phase is bainite. The second one (red dashed line, t2) implies a fast transformation like one for martensite. Therefore, the transformed phase at this stage is martensite.

Table S1. Process conditions for Steel E and F in sequential order.

Step	Steel E	Steel F
1	Hold for 10 minutes at 850°C	Hold for 10 minutes at 850°C
2	Cool down at a rate of 100°C per second	Cool down at a rate of 50°C per second

Table S2. Pictorial description of input features.

Input feature	Description
Grain size	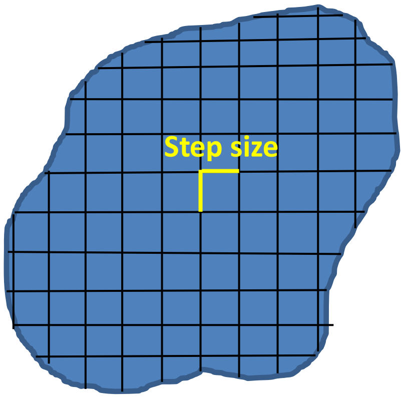 Distribution of various grain size.
Grain average misorientation (GAM)	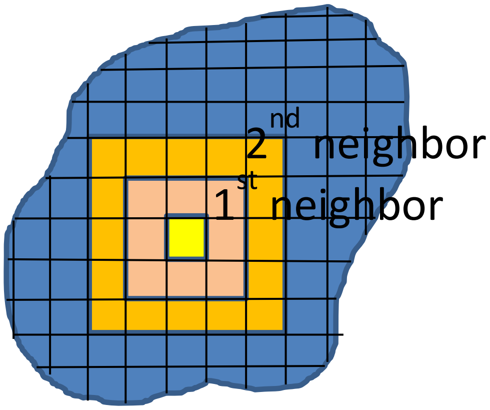 Comparative degree of grain deformation. The average misorientations of neighboring points (in this study, 1st neighbor) are considered.
Grain orientation spread (GOS)	

	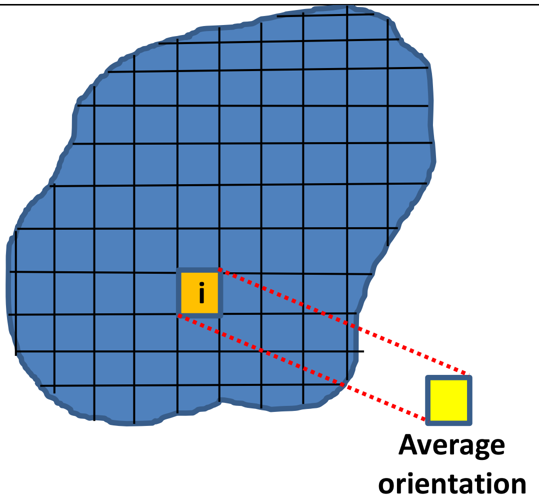<br>Average orientation of grains.
Area weighted average sharpness	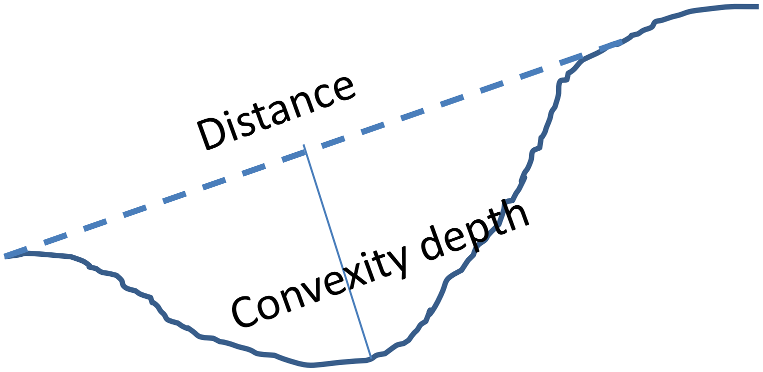<br>Depth and distance are defined as shown above.
